# The Hippo Signaling Pathway: The Trader of Tumor Microenvironment

**DOI:** 10.3389/fonc.2021.772134

**Published:** 2021-11-11

**Authors:** Duo Yang, Na Zhang, Meihua Li, Tao Hong, Wei Meng, Taohui Ouyang

**Affiliations:** ^1^ Department of the Forth Clinical Medical College of Nanchang University, Nanchang, China; ^2^ Department of Neurology, The First Affiliated Hospital of Nanchang University, Nanchang, China; ^3^ Department of Neurosurgery, The First Affiliated Hospital of Nanchang University, Nanchang, China

**Keywords:** Hippo pathway, cancer, molecular network, immune modulation, microenvironment

## Abstract

The Hippo pathway regulates cancer biology in many aspects and the crosstalk with other pathways complicates its role. Accumulated evidence has shown that the bidirectional interactions between tumor cells and tumor microenvironment (TME) are the premises of tumor occurrence, development, and metastasis. The relationship among different components of the TME constitutes a three-dimensional network. We point out the core position of the Hippo pathway in this network and discuss how the regulatory inputs cause the chain reaction of the network. We also discuss the important role of Hippo-TME involvement in cancer treatment.

## Introduction

Tumors are abnormal tissues caused by cell proliferation under the action of carcinogenic environmental factors. The tumor microenvironment (TME) includes various components, such as blood vessels, immune infiltration, fibroblasts, and the extracellular matrix. The interaction between tumor cells and TME may lead to tumor occurrence, metastasis and drug resistance. To explore the specific mechanism between them further is of great significance for the development of effective treatment methods. So far, Hippo pathway has been proved not only to control the development of normal tissues, but also to participate in the occurrence, development and metastasis of cancer. The purpose of this review is to illustrate the role of Hippo pathway in TME that can indirectly act on tumors. The Hippo pathway generally plays a vital role in fibroblast activation, stem cell maintenance, extracellular matrix change, immune infiltration, and angiogenesis, which doesn’t depend on the typical receptor-ligand interaction mode. It also influences the secretion of numerous molecules that affects the development of tumors *via* cells in TME. More and more evidence show that the Hippo pathway is widely involved in the pro-and antitumor effects in different tumor populations. We discuss the interactions between the Hippo pathway and the other components of the TME, attempts to explain its mechanism of action on tumors, and explores effective treatment options that may be realized in the future.

## Hippo Pathway at a Glance

**Figure 1:**
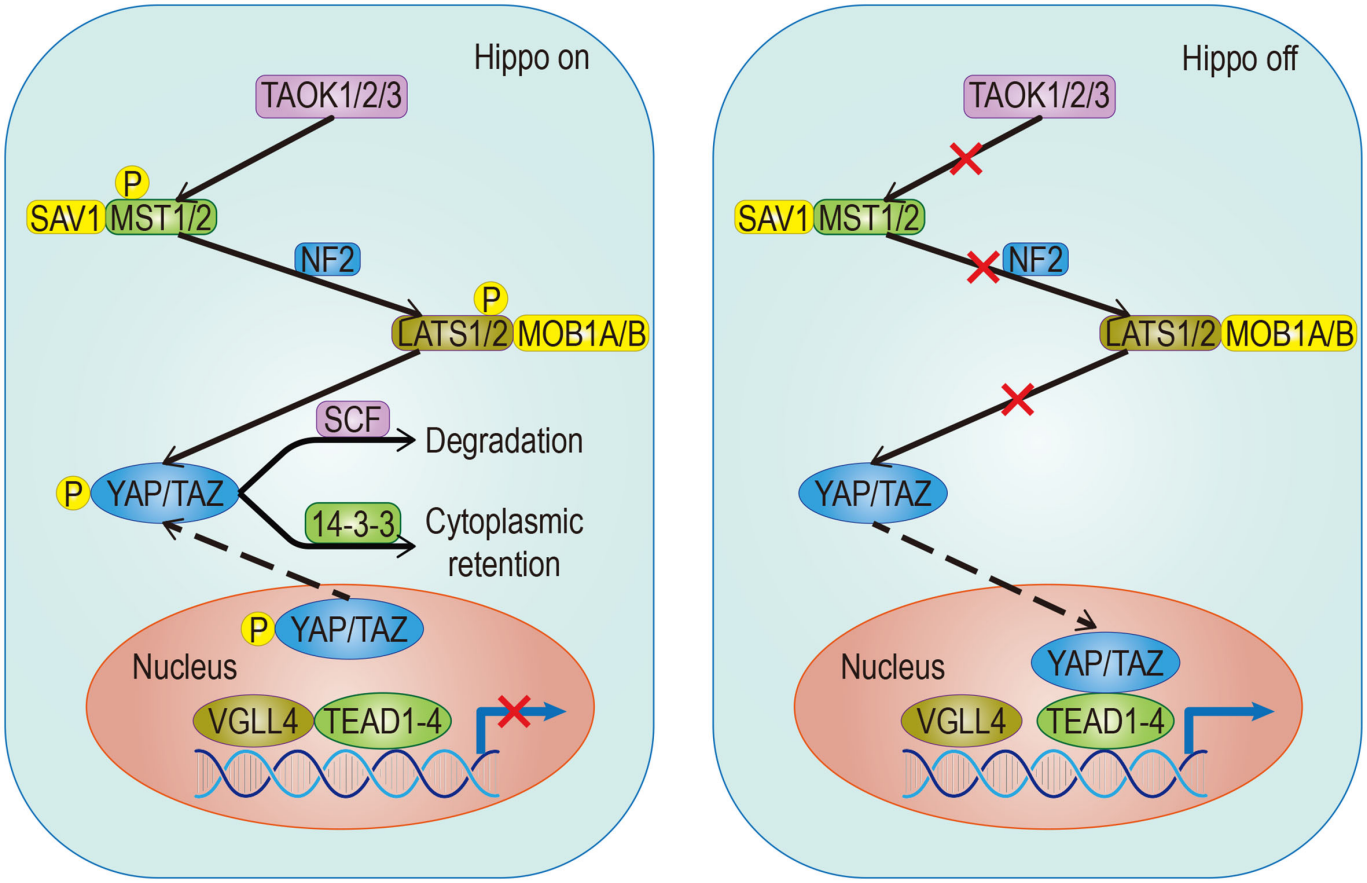
Classical signaling Hippo pathway. The target gene cannot be transcribed When the pathway is activated; Conversely, it can be transcribed when the pathway is suppressed. The solid arrow represents promotion, the dotted arrow represents the movement of components and the fork on the solid line represents the occlusion effect.

The Hippo pathway plays a central role in regulating organ size and maintaining dynamic tissue balance. The step of this mechanism is: First, TAOK1/2/3 phosphorylation, or MST1/2 automatic phosphorylation Initiates the Hippo kinase cascade; Second, activated MST1/2 phosphorylates LATS1/2; Third, activated LATS1/2 phosphorylates YAP/TAZ under the action of SAV1, MOB1A/B, and NF2; Finally, this results in the 14-3-3-mediated cytoplasmic retention and SCF-mediated degradation of YAP/TAZ (**Figure 1**). YAP/TAZ is a transcriptional coactivator that regulates gene transcription mainly by interacting with TEAD. In mouse models, the upregulation of TEAD target gene expression, with partial deletion of kinase cascade or YAP overexpression, can lead to increased progenitor cell proliferation and tissue overgrowth (1–3). Physical development is a very sophisticated process which follows strict protocol according to an organism’s genetic blueprint. The Hippo pathway plays a significant role in the drawing of this blueprint. Because of the Hippo pathway’s unique ability to promote regeneration, any abnormality of its core components, especially YAP/TAZ, is of great significance in promoting the migration, invasion, and malignancy of cancer cells. Aberrant overexpression of YAP/TAZ in tumors promotes tumorigenesis and is therefore considered an oncogene in a large number of solid cancers. Also, YAP/TAZ can enhance death-resistant and drug-resistant qualities of the cancer cell, which can be used as the premise of expansion of cancer stem cells (CSC) (4, 5). Consistent results in functional studies have revealed that the expression level of YAP may be associated with invasiveness of clinicopathological features and poor clinical outcomes (6, 7). For example, there is an excellent correlation between the predicted results and the actual results in patients with breast cancer, which follows the conclusion outlined above (4, 8). A special statement is needed that the regulatory effects of the Hippo pathway on tumors are highly dependent on the tumor environment. For example, in a mouse model of colorectal cancer, YAP/TAZ suppressed tumor growth by inducing reprogramming of cancer stem cells (9), suggesting a duality of the Hippo pathway. The Hippo pathway is regulated by a network containing multiple upstream regulators such as cell polarity, cell junctions, mechanical signals, and soluble factors that act through G protein-coupled receptors (GPCRs). In addition, the discovery that YAP/TAZ can crosstalk with other signaling pathways such as AMPK, Wnt, TGF-β, and Notch to control cell fate further adds to the complexity (10). Just as the Hippo signaling pathway has different effects on different organs, it has also been shown that the importance of activated YAP/TAZ in promoting tumors varies in different environments (11). Although YAP/TAZ activation seems to be a common feature of many tumors, differences in the forces driving activity and the modes of interaction are evident. Therefore, an in-depth understanding of the differences can help predict the prognosis of patients and guide the development of YAP/TAZ-targeted therapy.

## Hippo Pathway and Cancer-Associated Fibroblasts

Cancer-associated fibroblasts (CAFs), as the main cellular component of TME, are characterized by the expression of activation markers such as fibroblast activation protein, α-smooth muscle actin (α-SMA), and some secretory factors which are involved in immune invasion recruiting and extracellular matrix remodeling (12, 13). Indeed, CAFs are involved in the initiation, progression, and metastasis of disease (14). Now, the importance of Hippo signaling in regulating fibroblast activation in TME has been well demonstrated. In prostate cancer, YAP1 converts normal fibroblasts into CAFs by regulating SRC to activate cytoskeletal proteins and actin (15). In CAFs, YAP affects the expression of cytoskeleton regulatory factors such as ANLN, CTGF, and DIAPH3 (16). Through MYL9/myosin light chain-2, YAP can also affect actomyosin contractility and ECM remodeling (17). The fact that YAP/TAZ can enhance the function of CAFs by affecting the progression of matrix remodeling further confirms their role (12, 13). However, two particular points that need to be explained: One, for CAFs, depletion of YAP has more obvious influence than the depletion of TAZ (17), indicating that the downstream component of the signaling pathway which mediated by YAP is not only TAZ; Two, in breast cancer, not only the activity of MST1/2 doesn’t weaken in CAFs, but also the activity of LATS kinases and phosphorylated YAP increases (17), indicating that activation mode of YAP in CAF is different from the typical model.

CAFs directly interact with cancer cells and change the microenvironment by regulating paracrine signals mediated by inflammatory cytokines (12). Due to the interaction with interleukin in CAF, YAP/TAZ can affect the TME (11). In the stroma of both adenoma and carcinoma lesions, YAP nuclear accumulation has been observed. Also, the activation of YAP can be promoted in the stroma of the surrounding tumor area of advanced cancer (18).

Serum Response Factor (SRF) plays an essential role in fibroblasts activation during vertebrate development. Myocardin-related transcription factor (MRTF) is coactivator of SRF. Activation of CAFs is promoted when YAP-TEAD cooperates with MRTF-SRF (19). Moreover, YAP/TEAD and MRTF-SRF can also activate each other by modulating actin cytoskeletal dynamics in CAFs (16). Because of the unknown communication between MRTF-SRF and YAP/TAZ-TEAD in different types of cancer *in vivo* and the necessity of CAF for chemotherapy, there is still further research to be done in the future.

## Hippo Pathway and Cancer Stem Cells

Cancer stem cells (CSCs) or tumor-initiating cells (TICs) have self-renewing and tumor-initiating characteristics that are present in at least some tumors and can lead to a heterogeneous spectrum of cancer cells (20, 21). The characteristics of CSCs are modulated by epithelial-mesenchymal transformation-induced transcription factors (EIFs) and endothelial cells (22). In addition to playing an essential role in drug resistance, metastasis, recurrence, and cancer mortality, CSCs can also influence the microenvironment by generating endothelial-like cells and promoting tumor vascularization (23). In a word, CSCs are closely related to cancer progression. To effectively carry out targeted antitumor therapy, it is necessary to investigate the mechanisms and characteristics of CSC signal transduction.

In addition to aberrant activation or mutation in stemness genes, the Hippo pathway is also related to cancer progression (24). Accumulating evidence suggests that the Hippo pathway influences early tumorigenesis by regulating the proliferation and maintenance of stem cells (SCs) to participate in cellular reprogramming and regulating tissue size (25, 26). YAP has been confirmed to have roles in establishing the TME, maintaining the homeostasis of normal tissue SCs, maintaining the pluripotency of embryonic stem cell and CSC pools, and promoting epidermal development and proliferation of SCs and progenitor cells (26–28).

Since the proliferation and survival of CSCs are related to lipid metabolism, and the Hippo signal participates in the lipid desaturation of CSCs, it is theoretically possible to achieve targeted clearance of CSCs *via* the Hippo signal (21). However, the role of YAP and TAZ plays in CSCs could be specified in a different environment and different signaling pathways (29). Therefore, further research is needed. Studies have shown that the Hippo and Wnt pathways are closely related in terms of regulation and function. The crosstalk between them contributes to the stem cell self-renewal functions (30), especially in intestinal homeostasis. For example, in the mammalian intestine, YAP1 can maintain crypt SCs proliferation and stemness by activating the Wnt signaling pathway (28). The crosstalk between the Hippo signal and other pathways which affect CSCs is complex, and the related mechanism remains to be studied.

## Hippo Pathway and Extracellular Matrix

**Figure 2:**
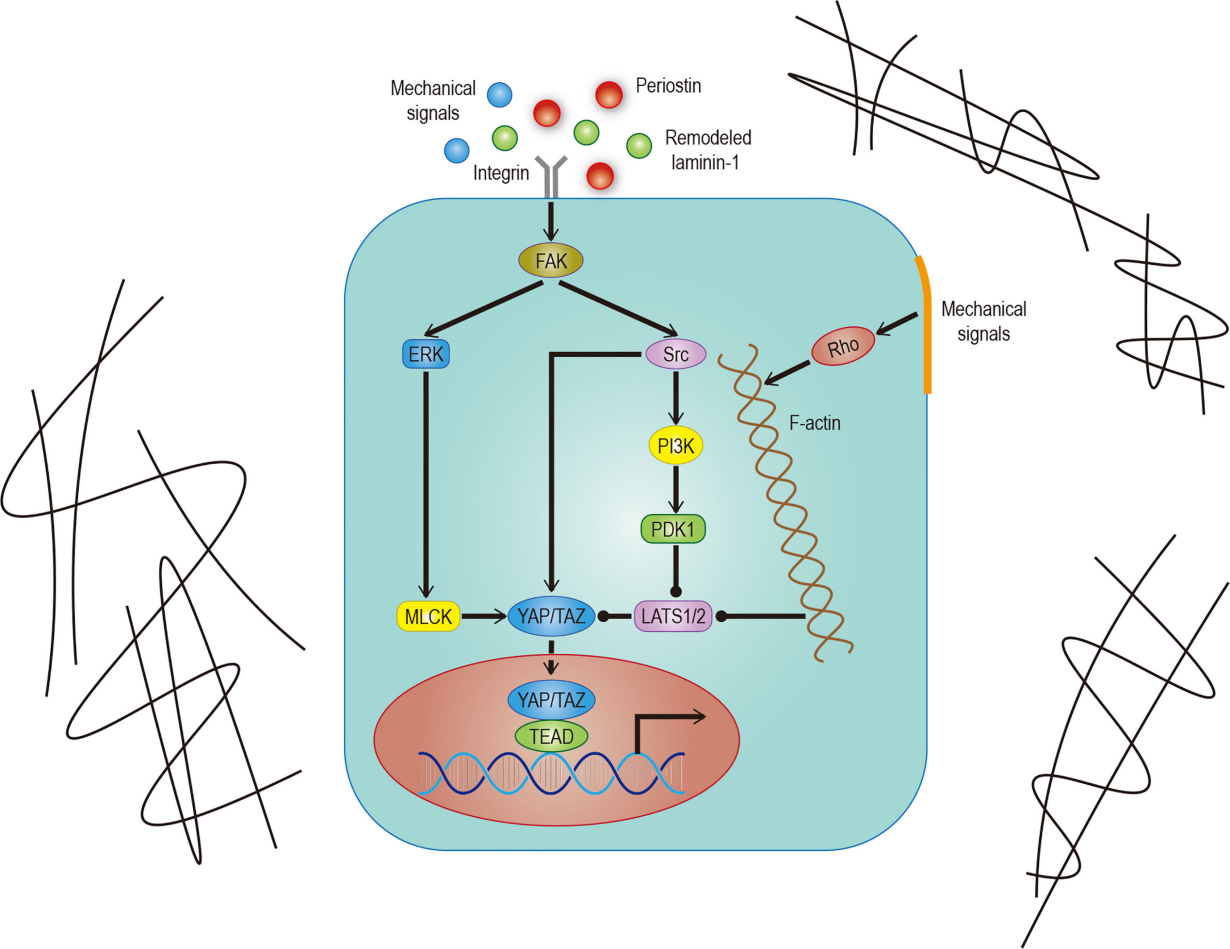
Hippo signaling in ECM. Effects of different mechanical signals mediated by integrins or the cytoskeleton on YAP/TAZ. The effect of remodeled laminin-1 on YAP mediated by FAK-ERK-MLCK has been confirmed. Although it is very likely to exist, the effect on TAZ remains to be studied. The solid arrow represents promotion, the solid dot represents inhibition.

In addition to soluble signals, cells can also perceive their microenvironment through mechanical cues derived from the extracellular matrix (ECM). Some studies point out that Hippo signals are nuclear relays that link mechanical signals to nuclear transcriptional signals (31). For example, cells grown in the ECM with different hardness show different YAP localizations (32, 33). Integrin plays an important role in ECM signal transduction which is mediated by Hippo signals (33). Recent studies have found that the ECM hardness can inhibit Salvador (Sav)–LATS association by regulating the integrin-mediated focal adhesion kinase (FAK)–Src–PI3K–PDK1 pathway, which in turn inhibits the Hippo signal pathway (34, 35). Src can also promote YAP activity by phosphorylating LATS or directly phosphorylating YAP (36, 37). Not only that, the regulation of dormant cancer cell awakening by remodeled laminin-1 and the promotion of colorectal tumorigenesis by fibroblast secreted periostin support the notion that the ECM is able to regulate YAP/TAZ (38, 39) (**Figure 2**).

Regulation of the Hippo pathway by mechanical upstream signals, such as extracellular matrix stiffness, cell shape, and cell density, largely depends on the effects of mechanical signals on the F-actin cytoskeleton, tension within the cytoskeleton, tension on cell-cell and cell ECM matrix attachments (40). Cytoskeleton tension is produced by myosin, and its contraction level is usually proportional to the activity of YAP (35). One of the critical regulators of the myosin cytoskeleton is the Rho-Rock (Rho-associated protein kinase)-MLC (non-myosin II light chain) pathway. Similar changes in YAP/TAZ activity can also be caused by manipulating a special part of the pathway (33, 41). Inhibiting the key cytoskeleton regulatory factor Rho can inhibit the effects of the cytoskeleton-mediated mechanical signals on the Hippo signal (33, 42). It is important to note that other non-mechanical upstream inputs of the Hippo pathway may also affect Hippo signals through cytoskeleton regulation (43). There is also a Rho-independent mechanism for the regulation of mechanical signals on the Hippo pathway (44). Another spectrin based cytoskeleton can also regulate Yorkie (Yki)/YAP activity but is functionally opposite to the F-actin cytoskeleton (45, 46).

Aberrant crypt foci (ACF) are the earliest sign of morphological damage in the process of adenoma formation. Researchers have speculated that the activation of YAP in ACF might be due to the continuous change of shape and increasing hardness of the lesions during the growth process (47). In intestinal organ culture, high ECM hardness can promote the survival of YAP-dependent intestinal stem cells (ISC) (48, 49), indicating that the mechanical stimulation of YAP due to increased ECM hardness may play an essential role in the development of colorectal cancer. There is no doubt that further exploration of its mechanism is helpful for targeted therapy.

## Hippo Pathway and Tumor Immunity

According to the latest understanding, immune infiltration composed of all immune cells involved in TME is defined as the main factor regulating tumor development. Studies have shown that CD8+T cells are immune infiltrated in a variety of solid tumors, suggesting that the density of CD8+T cells is closely related to the prognosis of solid tumors, such as colorectal cancer and breast cancer (50). However, the impact of this infiltration on tumors is not absolute: phenomena related to transcriptional signature of type I interferons indicate that innate immunity is activated, and phenomena related to the regulation of T cells (Treg) and myeloid-derived suppressor cells (MDSCs) indicate that immune responses are suppressed. For cancer cells, some of the infiltrating immune cells could destroy them while others could tolerate them (51, 52).

In tumor immunology, T cells are essential for both immune escape and antitumor immunity (53, 54). As the essential component of tumor immunity, YAP/TAZ shows an immunomodulatory effect by regulating immune cells functions. However, accumulated evidence indicates that atypical rather than typical Hippo signaling plays a crucial role in regulating T cells activity (55). For example, the homeostasis and priming of CD8+T cell which mediated by DC require the selective orchestration of Mst1/2 (56). YAP and TAZ have different regulatory effects on different immune cell subsets that play different roles in tumor immunity. The main mechanism of tumor immune surveillance in the TME is CD8+ cytotoxic T cells response, while Tregs with CD4+ CD25+ infiltration could inhibit T cells activity and ulteriorly promote tumor progression (54, 57). Th17 is a subset of T helper lymphocytes that can drive antitumor immune responses by activating effector CD8+ T cells. TAZ can promote the differentiation of TH17 and inhibit the differentiation of Tregs (58). The mechanism is to induce the definition transcription factor RORγ T in Th17 cells and promote the degradation of the Treg cell master regulator Foxp3, which is critical for TH17 and Tregs respectively (58). As for YAP, according to the analysis of glandular tissues of liver cancer, melanoma, and stomach cancer, it can be found that YAP can promote the differentiation of Treg (59, 60). This function is realized by upregulating the activin signaling, which enhances TGFβ/SMAD activation in Tregs (60). Also, there is a positive relationship between tumoral YAP expression and Tregs infiltration (59). All in all, TAZ promotes antitumor immunity while YAP promotes immune escape.

As the most important and highly infiltrated cells in the TME, tumor associated macrophages (TAMs), mainly including M1 and M2 phenotypes, play a crucial role in tumor immunity. According to the condition of TME, TAMs can be polarized into different phenotypes: M1 type TAMs promote inflammation by secrete IL-1, TNF-αand INOS; M2 type TAMs resolves inflammation by secrete IL-10, IL-4, arginase-1 (arg-1) (61, 62). In a word, one is antitumoral and another one is protumoral. It is known that there is a strong correlation between recruitment of TAMs and poor prognosis of various tumor types such as lung, hepatic, and colon cancers (63, 64). This phenomenon isn’t solely due to the difficulty of macrophages to polarize to M1 phenotype, considering that the Toll-like receptor (TLR) in TME is not activated (65, 66), and the Hippo-YAP signaling also plays a regulatory role in the recruitment and polarization of TAMs, especially for M2. By influencing several cytokines and chemokines, secreted by tumor cells such as IL-6, CCL2, and CSF-1 (67–69), YAP recruits the TAMs, remodels the composition of TME, and promotes the development of tumors. In human monocytic cells (THP-1), YAP-silencing only reduces the expression level of M2 markers (70); In hepatocytes, YAP-activating only promotes the polarization of TAMs into M2 phenotype (67); In pancreatic ductal adenocarcinoma (PDAC), YAP-deleting only promotes the polarization of TAMs into M1 phenotype (71); In colon cancer cells, down-regulation of YAP expression is not only inhibiting tumor development but also reducing the expression of IL-4 and IL-13 (70), which are vital inducers of M2 polarization, thereby inhibiting the polarization of TAMs into M2 phenotype. It should be noted that myeloid-derived suppressor cells (MDSCs), which represent phenotypically heterogeneous immature myeloid cells, can also promote tumorigenesis by inhibiting T cells activity, especially CD8+ cytotoxic T cells (52). The regulatory effect of YAP on MDSCs is reflected in two aspects: First, directly transcribing chemokine CXCL5 to promote the recruitment of MDSCs in prostate tumor cells (72); Second, promoting the expression of IL-6, CSF1-3, TNF-α, IL-3, CXCL1/2, and CCL2 to promote the recruitment and systematic differentiation of MDSCs in the Kras: p53-mutant PDAC model (71). In sum, YAP can promote tumorigenesis and tumor immune escape (**Table 1**).

**Table 1:** Effects of regulatory factors from different sources on immune cells.

Immune cell type	Source of regulating factor	Regulating factor type	Effect
M1	Tumor cells	IL-6, CCL-2, CSF-1	Recruitment
		IL-6	Polarization
	Itself	IL-1, TNF-α, INOS, IL-6	Affect the tumor
M2	Tumor cells	IL-6, CCL-2, CSF-1	Recruitment
		IL-4, IL-13	Polarization
	Itself	IL-4, IL-10, Arg-1, TGF-β	Affect the tumor
MDSCs	Tumor cells	CXCL5, IL-6, CSF1-3, TNF-α, IL-3, CXCL1/2, CCL-2	Recruitment
		IL-6, CSF1-3, TNF-α, IL-3, CXCL1/2, CCL-2	Polarization
	Itself	NO, ROS, TGF-β	Affect the tumor

Both Recruitment and Polarization refer to the effect of regulatory factors on immune cells; Itself refers to the corresponding immune cells. The contents of the table come from five articles (67, 72–75).

**Figure 3:**
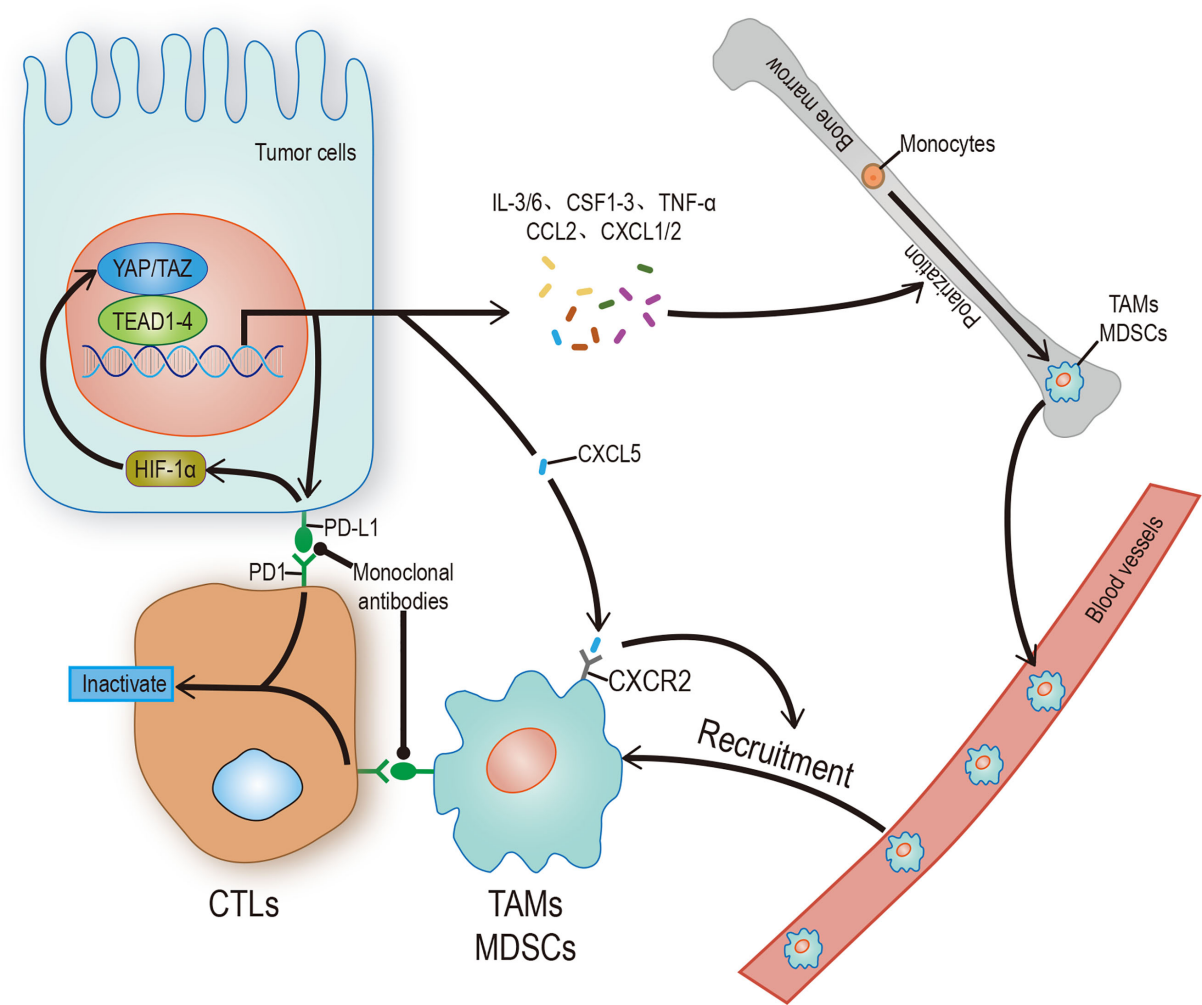
Hippo signaling in tumor immunity. YAP/TAZ promotes the transcription of target genes, and its products include regulatory factors and receptors, which can promote the polarization of immune cells, recruit and enhance the stability of YAP/TAZ-TEAD complex, and finally inhibit the activity of CTLs. CTLs can be restarted by blocking the binding of receptor ligands. The solid arrow represents promotion, the solid dot represents inhibition.

Immune escape is both a challenge and an opportunity. PD-L1 and CTLA-4, often used as tumor cell surface markers, can reduce the proliferation and effector function of the cytotoxic T cell (CTL) by binding to homologous receptors on the CTL. Therefore, any factor that upregulates PD-L1 or CTLA-4, such as interaction with YAP/TAZ, can promote immune escape. On the one hand, YAP/TAZ could upregulate the level of PD-L1 by directly binding to the TEADs promoter in human cancer cells (76, 77). On the other hand, PD-L1 can increase the stability and activity of YAP/TAZ without affecting the Hippo pathway (78), indicates a positive feedback loop may exist. Based on the information above, people have successfully activated T cells in some tumor cells by using monoclonal antibodies which against PD-L1/CTLA-4 or PD-L1 receptors (**Figure 3**). Unfortunately, this method doesn’t affect most tumor cells due to the existence of other immunosuppressive mechanisms (79, 80). Therefore, a future research direction may concentrate on exploring practical ways to mediate T cell reactivation.

## Hippo Pathway and Tumor Vasculature

**Figure 4:**
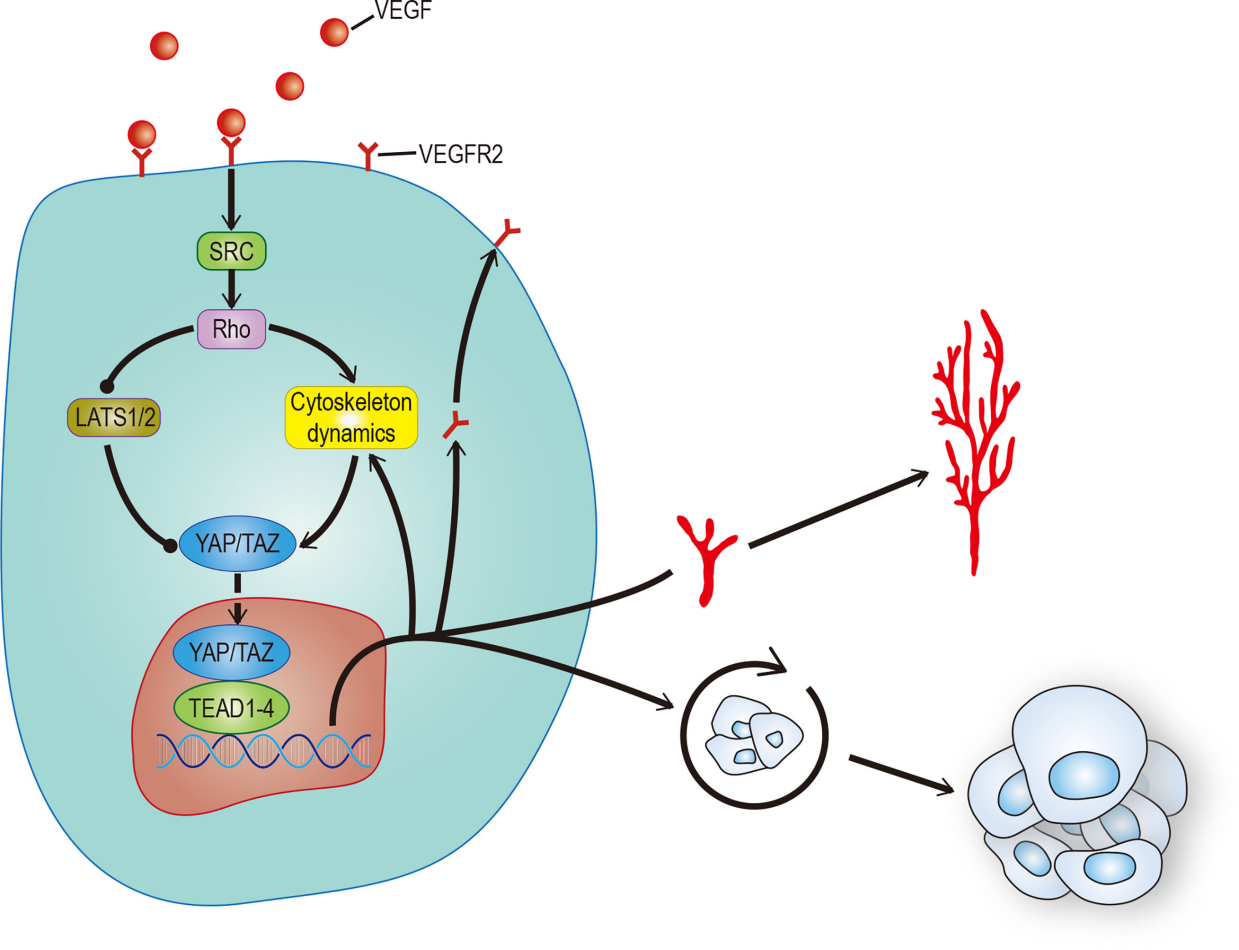
VEGF promotes angiogenesis and CSCs self-renewal. VEGF activates RhoGTPases through SRC, which in turn inhibits LATS or promotes cytoskeleton rearrangement to activate YAP/TAZ. The activation of YAP/TAZ can not only promote the movement of VEGFR to the cell membrane to form positive feedback, but also promote angiogenesis and self-renewal of tumor stem cells. The solid arrow represents promotion, the solid dot represents inhibition.

Angiogenesis plays an important role in both ontogeny and tumor progression. Most cancer patient deaths are caused by tumor metastasis. The main reason is that vascular mimicry increases the tendency of cancer cells to enter blood circulation (81). Still, the formation of new blood vessels in and around the tumor is more important. Angiogenesis is a complex process involving the dynamic changes of endothelial cells (EC). Recent studies have found that the Hippo pathway and its main effect factor YAP/TAZ, contribute to angiogenesis. One study showed that overactivation of YAP/TAZ led to excessive angiogenesis in mice (82), and another study showed that activation of YAP/TAZ mediated vascular endothelial growth factor (VEGF) signaling in various endothelial cells *in vitro* (83). There are a variety of signal pathways that regulate angiogenesis. To date, VEGF, especially vascular endothelial growth factor A (VEGF-A), is recognized as the most recognized pro-angiogenic signal. In addition to stimulating the development of endothelial cells, it can also stimulate tumor cells, especially cancer stem cells (CSCs), to affect the occurrence, development, and recurrence of tumors (84). This has stimulated great interest in exploring the regulatory role of VEGF in tumor cells and their potential use as therapeutic targets. VEGF is the main initiator and regulator of angiogenesis (85). It induces angiogenesis by binding to its receptors VEGFR1 and VEGFR2, thereby regulating the proliferation, survival, and migration of endothelial cells (86). Among these receptors, the VEGF-VEGFR2 signal axis needs the participation of YAP/TAZ (82, 83). Some studies have found that YAP/TAZ can regulate angiogenesis mediated by VEGF through regulation of the circulation of VEGFR2 on the cell surface (82, 83). Other studies found that VEGF increases the nuclear localization of YAP and that Verteporfin significantly reduced VEGF-induced angiogenesis, suggesting that YAP/TAZ play an important role in VEGF-induced angiogenesis *in vivo* (87). There may be many VEGF mechanisms that activate YAP/TAZ. Src family kinases activated by VEGFR2, and cytoskeletal rearrangement mediated by Rho family GTPases activated by Src family kinases may be the core (88) (**Figure 4**). All the evidence outlined above suggests that YAP/TAZ plays an important role in promoting the feedforward loop of angiogenesis. Not only does the Hippo-YAP/TAZ signal acts as a new influencing factor, but also its crosstalk with other signal pathways such as Notch, Wnt, TGF, BMP, and GPCR helps us to understand angiogenesis further (89, 90). This provides a new perspective to explain physiological angiogenesis and in turn helps design new therapeutic targets for pathological angiogenesis.

## Hippo Pathway Crosstalk With Other Pathways

**Figure 5:**
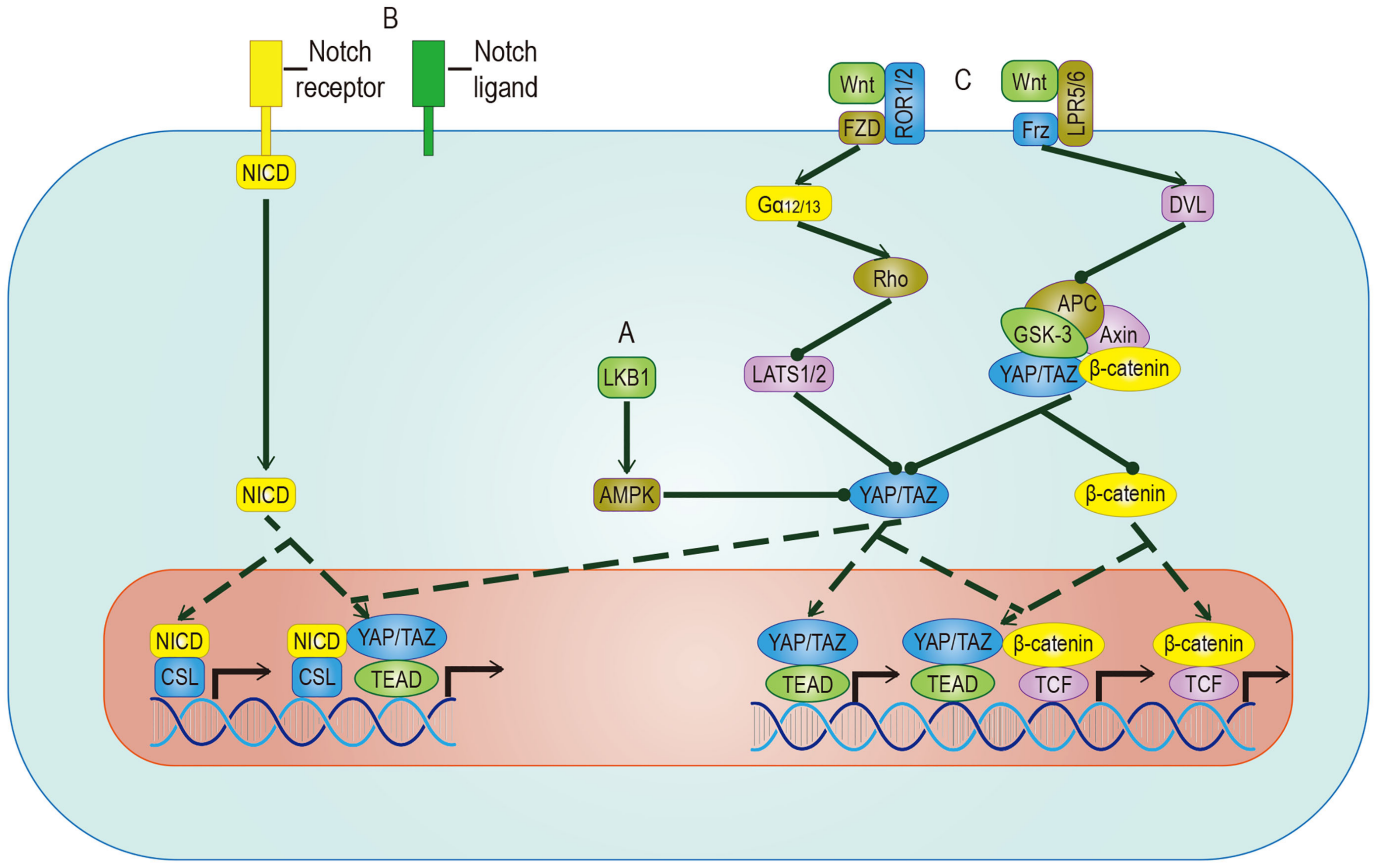
Crosstalk between YAP/TAZ and other pathways. **(A)** AMPK mediates the inhibitory effect of LKB1 on YAP/TAZ, but whether AMPK can inhibit YAP/TAZ through LATS remains to be studied. **(B)** YAP/TAZ can promote the expression of Notch receptor and Notch ligand. Once Hippo and Notch signaling are activated at the same time, they not only regulate the expression of common target genes but also facilitate the nuclear translocation of NICD and YAP/TAZ. **(C)** The target genes regulated by YAP/TAZ have different effects on different Wnt pathways. In Wnt-ROR1/2-FZD pathway, the activity of YAP/TAZ is enhanced by inhibiting LATS. In Wnt-LPR5/6-Frz pathway, by promoting the detachment and nuclear translocation of YAP/TAZ from the destructive complex, YAP/TAZ and accumulated β-catenin regulate the expression of common target genes. YAP/TAZ target genes include Notch receptor/ligand and Wnt5a/b, which regulate Notch pathway and Wnt pathway respectively. The solid arrow represents promotion, the dotted arrow represents the movement of components, and the solid dot represents inhibition.

The Hippo signal is related to various molecular inputs, including other signaling pathways (**Figure 5**). To find more efficient targets for cancer treatment, it is necessary to explore these links, which are outlined below.

### AMPK Pathway

AMP-activated protein kinase (AMPK) is a crucial energy receptor that regulate cell growth and metabolism by determining the energy status of the cell through monitoring the AMP: ATP ratio. Studies have shown there is a relationship between AMPK and the Hippo pathway. It is well known that the Hippo pathway can regulate cell activity by sensing the energy state, and it has been established that AMPK can regulate the activity of YAP. However, there is some controversy about the mechanism. Among the proposed mechanisms, it is accepted that AMPK phosphorylates YAP directly in a way parallel to the Hippo pathway. However, different studies have proposed opposing theories to address the question of whether AMPK regulates the activity of YAP by activating LATS, indicating that the relationship between them requires more research (91, 92). While it is known that energy stress can activate AMPK and Hippo to phosphorylate YAP at different sites in response to the energy stress, it is important to mention glucose-transporter 3 (GLUT3) in this discussion. YAP and GLUT3 are highly expressed in different human cancers, and their expressions in tumor samples are positively correlated. YAP promotes glucose metabolism by upregulating the expression of GLUT3, suggesting that YAP may lead to cancer by stimulating glucose uptake and glycolysis (92). This discovery of AMPK as a YAP kinase expands the understanding of the classic Hippo pathway.

### Notch Pathway

The biological effects of activated YAP/TAZ are to promote cell growth and inhibit differentiation. A sizeable part of the inhibition of differentiation is achieved by the Notch pathway. YAP/TAZ and Notch cascades interact mainly in two ways: one is transcriptional regulation of Notch receptors or ligands mediated by YAP/TAZ, the other one is the expected target gene of YAP/TAZ and NICD transcriptional regulation. In epidermal cells, YAP/TAZ drives the expression of the Notch ligand, mediates the cis-inhibition of cells, and maintains an undifferentiated state. On the contrary, YAP/TAZ gene knockout shuts down the expression of the Notch ligand and releases a Notch signal, which leads to cell differentiation (93). In most studies on epidermal stem cells, Notch is located downstream of YAP/TAZ. However, in a report on the liver, YAP is placed downstream of Notch (94), which means that the RBPJ/NICD complex plays a transcriptional inhibitory role in YAP, suggesting that there may be two-way crosstalk between them. In recent studies of human embryonic rhabdomyosarcoma (ERMS), this two-way crosstalk is described (95). In human ERMS tumors and rhabdomyosarcoma-derived cell lines, both YAP/TAZ and Notch signaling are hyperactivated (96, 97). Two assumptions have been put forward to explain this phenomenon: One is that Notch signaling up-regulates the level of YAP at the transcriptional level, and the up-regulated YAP up-regulates Notch signaling by up-regulating Notch ligands and transcription factors (97); The second is that YAP/TAZ maintains the undifferentiated state by cis-inhibiting Notch signaling and induces the proliferation of adjacent tumor cells by transactivating Notch signaling (11). In a clinical study, researchers proposed a more accurate explanation based on the above assumptions: YAP/TAZ promotes Notch signaling transduction by activating the expression of Jag1. At the same time, Notch signaling inhibits β-Trcp-mediated degradation through NICD and stabilizes the TAZ protein in a way that does not involve the Hippo pathway (89). In summary, there is a positive feedback loop between YAP/TAZ and Notch signaling.

### Wnt Pathway

Wnt proteins control body development and tissue homeostasis by binding to different receptors. Two of its working ways are mentioned here, namely the classic Wnt/β-catenin pathway and alternative Wnt pathway. In the alternative Wnt pathway, YAP/TAZ is the critical medium. The exact Wnt-YAP/TAZ signal transduction pathway has been identified. Interestingly, Wnt5a/b ligands are both upstream activators and downstream target genes of YAP/TAZ-TEAD, suggesting the existence of potential positive feedback loops. In addition, another critical role of the alternative Wnt pathway is to antagonize the Wnt/β-catenin signal, which is realized by the YAP/TAZ-TEAD-induced secretion of the Wnt/β-catenin signal inhibitor (98). In the Wnt/β-catenin pathway, YAP/TAZ, as a component of the destructive complex, dissociated from the destructive complex, enters the nucleus and forms a complex with accumulated β-catenin, which promotes the transcriptional activation of a group of target genes such as SOX2、SNAI2、BCL2L1 and BIRC5 (99), cooperatively inducing cell proliferation and promoting tumorigenesis. A range of evidence supports these effects: The nuclear localization of β-catenin and YAP/TAZ is found in nearly 80% of hepatoblastoma tissues, and the expression level of both is related to the growth rate of tumor (100); The expression of β-catenin and YAP/TAZ is considered to be a necessary condition for the growth of colorectal cancer and LKB1 inhibits the nuclear translocation of both to inhibit the proliferation of gastric cancer (101). In intestinal tumorigenesis, some genes such as Myc and CyclinD1 have been proven to be downstream of the Hippo signaling pathway and the Wnt/β-catenin signaling pathway, which are involved in the regulation of cell cycle and proliferation (102, 103). The ubiquitination of the Wnt and Hippo pathways’ component participates in maintaining CSC stemness (104). A series of trials using both of them as therapeutic targets showed obvious results. We believe that a more in-depth study of the crosstalk between Wnt and YAP/TAZ will help to improve the understanding of tumorigenesis.

## Discussion

In this review, we explore the role of YAP/TAZ in some aspects of tumorigenesis, development, and metastasis. The widespread activation of YAP/TAZ in human cancer indicates that in addition to some of the relationships mentioned above, the upstream MST1/2 kinase and LATS1/2 kinase, the downstream binding factor TEADs, YAP/TAZ itself, and the interaction between YAP/TAZ and TEADs may be exploited as targets for cancer therapy. Although some experiments have shown that Verteporfin and VGLL4 mimic peptides that inhibit tumor growth by inhibiting YAP/TAZ transcriptional activity in YAP/TAZ-dependent mouse tumor models, there is also a recent, exciting finding that inhibiting TEADs palmitoylation is likely to block YAP-dependent transcription (105). However, the extensive role of the Hippo pathway *in vivo* is limited. The consequences of the hasty use of YAP/TAZ inhibitors can be catastrophic. As YAP/TAZ has a powerful ability to promote regeneration, including myocardial regeneration, the development of related drugs such as LATS inhibitors is likely to be of great significance in promoting wound healing, tissue repair, and regeneration. It should be noted that the treatment of artificially activated YAP/TAZ for repair is likely to be accompanied by specific risks to the tumor, so extra caution is essential. Future research is required to explore the mechanism of the Hippo pathway further, and advanced in technology will provide a more comprehensive understanding towards the goal of making treatment of the Hippo pathway, a feasible target in cancer treatment.

## Author Contributions

DY, NZ, ML, and TH composed the manuscript while WM and TO conceived of the review subject matter and provided editorial review. All authors contributed to the article and approved the submitted version.

## Funding

The present study was supported by the National Natural Science Foundation of China (grant no. 81760447; grant no. 81960247, Project of Science and Technology Department of Jiangxi Province (grant no. S2019QNJJB1056; grant no. 20202BABL206099 and Jiangxi Provincial Education Department Project (grant no. GJJ180054; grant no. GJJ180116).

## Conflict of Interest

The authors declare that the research was conducted in the absence of any commercial or financial relationships that could be construed as a potential conflict of interest.

## Publisher’s Note

All claims expressed in this article are solely those of the authors and do not necessarily represent those of their affiliated organizations, or those of the publisher, the editors and the reviewers. Any product that may be evaluated in this article, or claim that may be made by its manufacturer, is not guaranteed or endorsed by the publisher.

## References

[1] Chen Q Zhang N Xie R Wang W Cai J Choi KS. Homeostatic Control of Hippo Signaling Activity Revealed by an Endogenous Activating Mutation in YAP. Genes Dev (2015) 29(12):1285–97. doi: 10.1101/gad.264234.115 PMC4495399 26109051

[2] Nishio M Hamada K Kawahara K Sasaki M Noguchi F Chiba S. Cancer Susceptibility and Embryonic Lethality in Mob1a/1b Double-Mutant Mice. J Clin Invest (2012) 122(12):4505–18. doi: 10.1172/JCI63735 PMC3533542 23143302

[3] Zhang N Bai H David KK Dong J Zheng Y Cai J. The Merlin/NF2 Tumor Suppressor Functions Through the YAP Oncoprotein to Regulate Tissue Homeostasis in Mammals. Dev Cell (2010) 19(1):27–38. doi: 10.1016/j.devcel.2010.06.015 20643348 PMC2925178

[4] Bartucci M Dattilo R Moriconi C Pagliuca A Mottolese M Federici G. TAZ Is Required for Metastatic Activity and Chemoresistance of Breast Cancer Stem Cells. Oncogene (2015) 34(6):681–90. doi: 10.1038/onc.2014.5 24531710

[5] Cordenonsi M Zanconato F Azzolin L Forcato M Rosato A Frasson C. The Hippo Transducer TAZ Confers Cancer Stem Cell-Related Traits on Breast Cancer Cells. Cell (2011) 147(4):759–72. doi: 10.1016/j.cell.2011.09.048 22078877

[6] Yeo MK Kim SH Kim JM Huang SM Kim MR Song KS. Correlation of Expression of Phosphorylated and Non-Phosphorylated Yes-Associated Protein With Clinicopathological Parameters in Esophageal Squamous Cell Carcinoma in a Korean Population. Anticancer Res (2012) 32(9):3835–40. 22993326

[7] Zhao J Li X Yang Y Zhu D Zhang C Liu D. Effect of YAP1 Silencing on Esophageal Cancer. Onco Targets Ther (2016) 9:3137–46. doi: 10.2147/OTT.S102338 PMC4888714 27307755

[8] Diaz-Martin J Lopez-Garcia MA Romero-Perez L Atienza-Amores MR Pecero ML Castilla MA. Nuclear TAZ Expression Associates With the Triple-Negative Phenotype in Breast Cancer. Endocr Relat Cancer (2015) 22(3):443–54. doi: 10.1530/ERC-14-0456 25870251

[9] Cheung P Xiol J Dill MT Yuan WC Panero R Roper J. Regenerative Reprogramming of the Intestinal Stem Cell State *via* Hippo Signaling Suppresses Metastatic Colorectal Cancer. Cell Stem Cell (2020) 27(4):590–604.e9. doi: 10.1016/j.stem.2020.07.003 32730753 PMC10114498

[10] Yu FX Meng Z Plouffe SW Guan KL. Hippo Pathway Regulation of Gastrointestinal Tissues. Annu Rev Physiol (2015) 77:201–27. doi: 10.1146/annurev-physiol-021014-071733 25293527

[11] Zanconato F Cordenonsi M Piccolo S. YAP/TAZ at the Roots of Cancer. Cancer Cell (2016) 29(6):783–803. doi: 10.1016/j.ccell.2016.05.005 27300434 PMC6186419

[12] LeBleu VS Kalluri R. A Peek Into Cancer-Associated Fibroblasts: Origins, Functions and Translational Impact. Dis Model Mech (2018) 11(4). doi: 10.1242/dmm.029447 PMC5963854 29686035

[13] Yoshida GJ Azuma A Miura Y Orimo A. Activated Fibroblast Program Orchestrates Tumor Initiation and Progression; Molecular Mechanisms and the Associated Therapeutic Strategies. Int J Mol Sci (2019) 20(9). doi: 10.3390/ijms20092256 PMC6539414 31067787

[14] Gascard P Tlsty TD. Carcinoma-Associated Fibroblasts: Orchestrating the Composition of Malignancy. Genes Dev (2016) 30(9):1002–19. doi: 10.1101/gad.279737.116 PMC4863733 27151975

[15] Shen T Li Y Zhu S Yu J Zhang B Chen X. YAP1 Plays a Key Role of the Conversion of Normal Fibroblasts Into Cancer-Associated Fibroblasts That Contribute to Prostate Cancer Progression. J Exp Clin Cancer Res (2020) 39(1):36. doi: 10.1186/s13046-020-1542-z 32066485 PMC7027236

[16] Noguchi S Saito A Nagase T. YAP/TAZ Signaling as a Molecular Link Between Fibrosis and Cancer. Int J Mol Sci (2018) 19(11). doi: 10.3390/ijms19113674 PMC6274979 30463366

[17] Calvo F Ege N Grande-Garcia A Hooper S Jenkins RP Chaudhry SI. Mechanotransduction and YAP-Dependent Matrix Remodelling Is Required for the Generation and Maintenance of Cancer-Associated Fibroblasts. Nat Cell Biol (2013) 15(6):637–46. doi: 10.1038/ncb2756 PMC3836234 23708000

[18] Yoshida GJ. Regulation of Heterogeneous Cancer-Associated Fibroblasts: The Molecular Pathology of Activated Signaling Pathways. J Exp Clin Cancer Res (2020) 39(1):112. doi: 10.1186/s13046-020-01611-0 32546182 PMC7296768

[19] Thompson BJ. YAP/TAZ: Drivers of Tumor Growth, Metastasis, and Resistance to Therapy. Bioessays (2020) 42(5):e1900162. doi: 10.1002/bies.201900162 32128850

[20] Ajani JA Song S Hochster HS Steinberg IB. Cancer Stem Cells: The Promise and the Potential. Semin Oncol (2015) 42(Suppl 1):S3–17. doi: 10.1053/j.seminoncol.2015.01.001 25839664

[21] Yi M Li J Chen S Cai J Ban Y Peng Q. Emerging Role of Lipid Metabolism Alterations in Cancer Stem Cells. J Exp Clin Cancer Res (2018) 37(1):118. doi: 10.1186/s13046-018-0784-5 29907133 PMC6003041

[22] Kaushal K Antao AM Kim KS Ramakrishna S. Deubiquitinating Enzymes in Cancer Stem Cells: Functions and Targeted Inhibition for Cancer Therapy. Drug Discov Today (2018) 23(12):1974–82. doi: 10.1016/j.drudis.2018.05.035 29864528

[23] Shibue T Weinberg RA. EMT. CSCs, and Drug Resistance: The Mechanistic Link and Clinical Implications. Nat Rev Clin Oncol (2017) 14(10):611–29. doi: 10.1038/nrclinonc.2017.44 PMC5720366 28397828

[24] Takebe N Harris PJ Warren RQ Ivy SP. Targeting Cancer Stem Cells by Inhibiting Wnt, Notch, and Hedgehog Pathways. Nat Rev Clin Oncol (2011) 8(2):97–106. doi: 10.1038/nrclinonc.2010.196 21151206

[25] Mo JS Park HW Guan KL. The Hippo Signaling Pathway in Stem Cell Biology and Cancer. EMBO Rep (2014) 15(6):642–56. doi: 10.15252/embr.201438638 PMC4197875 24825474

[26] Ramos A Camargo FD. The Hippo Signaling Pathway and Stem Cell Biology. Trends Cell Biol (2012) 22(7):339–46. doi: 10.1016/j.tcb.2012.04.006 PMC3383919 22658639

[27] Maugeri-Sacca M De Maria R. The Hippo Pathway in Normal Development and Cancer. Pharmacol Ther (2018) 186:60–72. doi: 10.1016/j.pharmthera.2017.12.011 29305295

[28] Hong AW Meng Z Guan KL. The Hippo Pathway in Intestinal Regeneration and Disease. Nat Rev Gastroenterol Hepatol (2016) 13(6):324–37. doi: 10.1038/nrgastro.2016.59 PMC5642988 27147489

[29] Barry ER Camargo FD. The Hippo Superhighway: Signaling Crossroads Converging on the Hippo/Yap Pathway in Stem Cells and Development. Curr Opin Cell Biol (2013) 25(2):247–53. doi: 10.1016/j.ceb.2012.12.006 23312716

[30] Fu V Plouffe SW Guan KL. The Hippo Pathway in Organ Development, Homeostasis, and Regeneration. Curr Opin Cell Biol (2017) 49:99–107. doi: 10.1016/j.ceb.2017.12.012 29316535 PMC6348871

[31] Mohri Z Del Rio Hernandez A Krams R. The Emerging Role of YAP/TAZ in Mechanotransduction. J Thorac Dis (2017) 9(5):E507–9. doi: 10.21037/jtd.2017.03.179 PMC5465147 28616323

[32] Dupont S Morsut L Aragona M Enzo E Giulitti S Cordenonsi M. Role of YAP/TAZ in Mechanotransduction. Nature (2011) 474(7350):179–83. doi: 10.1038/nature10137 21654799

[33] Zhao B Li L Wang L Wang CY Yu J Guan KL. Cell Detachment Activates the Hippo Pathway *via* Cytoskeleton Reorganization to Induce Anoikis. Genes Dev (2012) 26(1):54–68. doi: 10.1101/gad.173435.111 22215811 PMC3258966

[34] Kim NG Gumbiner BM. Adhesion to Fibronectin Regulates Hippo Signaling *via* the FAK-Src-PI3K Pathway. J Cell Biol (2015) 210(3):503–15. doi: 10.1083/jcb.201501025 PMC4523609 26216901

[35] Sun S Irvine KD. Cellular Organization and Cytoskeletal Regulation of the Hippo Signaling Network. Trends Cell Biol (2016) 26(9):694–704. doi: 10.1016/j.tcb.2016.05.003 27268910 PMC4993636

[36] Li P Silvis MR Honaker Y Lien WH Arron ST Vasioukhin V. alphaE-Catenin Inhibits a Src-YAP1 Oncogenic Module That Couples Tyrosine Kinases and the Effector of Hippo Signaling Pathway. Genes Dev (2016) 30(7):798–811. doi: 10.1101/gad.274951.115 27013234 PMC4826396

[37] Si Y Ji X Cao X Dai X Xu L Zhao H. Src Inhibits the Hippo Tumor Suppressor Pathway Through Tyrosine Phosphorylation of Lats1. Cancer Res (2017) 77(18):4868–80. doi: 10.1158/0008-5472.CAN-17-0391 28754671

[38] Albrengues J Shields MA Ng D Park CG Ambrico A Poindexter ME. Neutrophil Extracellular Traps Produced During Inflammation Awaken Dormant Cancer Cells in Mice. Science (2018) 361(6409). doi: 10.1126/science.aao4227 PMC6777850 30262472

[39] Ma H Wang J Zhao X Wu T Huang Z Chen D. Periostin Promotes Colorectal Tumorigenesis Through Integrin-FAK-Src Pathway-Mediated YAP/TAZ Activation. Cell Rep (2020) 30(3):793–806.e6. doi: 10.1016/j.celrep.2019.12.075 31968254

[40] Misra JR Irvine KD. The Hippo Signaling Network and Its Biological Functions. Annu Rev Genet (2018) 52:65–87. doi: 10.1146/annurev-genet-120417-031621 30183404 PMC6322405

[41] Cai J Song X Wang W Watnick T Pei Y Qian F. A RhoA-YAP-C-Myc Signaling Axis Promotes the Development of Polycystic Kidney Disease. Genes Dev (2018) 32(11-12):781–93. doi: 10.1101/gad.315127.118 PMC6049514 29891559

[42] Aragona M Panciera T Manfrin A Giulitti S Michielin F Elvassore N. A Mechanical Checkpoint Controls Multicellular Growth Through YAP/TAZ Regulation by Actin-Processing Factors. Cell (2013) 154(5):1047–59. doi: 10.1016/j.cell.2013.07.042 23954413

[43] Yu FX Zhao B Panupinthu N Jewell JL Lian I Wang LH. Regulation of the Hippo-YAP Pathway by G-Protein-Coupled Receptor Signaling. Cell (2012) 150(4):780–91. doi: 10.1016/j.cell.2012.06.037 PMC3433174 22863277

[44] Meng Z Qiu Y Lin KC Kumar A Placone JK Fang C. RAP2 Mediates Mechanoresponses of the Hippo Pathway. Nature (2018) 560(7720):655–60. doi: 10.1038/s41586-018-0444-0 PMC6128698 30135582

[45] Deng H Wang W Yu J Zheng Y Qing Y Pan D. Spectrin Regulates Hippo Signaling by Modulating Cortical Actomyosin Activity. Elife (2015) 4:e06567. doi: 10.7554/eLife.06567 25826608 PMC4412106

[46] Fletcher GC Elbediwy A Khanal I Ribeiro PS Tapon N Thompson BJ. The Spectrin Cytoskeleton Regulates the Hippo Signalling Pathway. EMBO J (2015) 34(7):940–54. doi: 10.15252/embj.201489642 PMC4388601 25712476

[47] Gregorieff A Wrana JL. Hippo Signalling in Intestinal Regeneration and Cancer. Curr Opin Cell Biol (2017) 48:17–25. doi: 10.1016/j.ceb.2017.04.005 28527754

[48] Crotti S Piccoli M Rizzolio F Giordano A Nitti D Agostini M. Extracellular Matrix and Colorectal Cancer: How Surrounding Microenvironment Affects Cancer Cell Behavior? J Cell Physiol (2017) 232(5):967–75. doi: 10.1002/jcp.25658 27775168

[49] Kai F Laklai H Weaver VM. Force Matters: Biomechanical Regulation of Cell Invasion and Migration in Disease. Trends Cell Biol (2016) 26(7):486–97. doi: 10.1016/j.tcb.2016.03.007 PMC4970516 27056543

[50] Fridman WH Zitvogel L Sautes-Fridman C Kroemer G. The Immune Contexture in Cancer Prognosis and Treatment. Nat Rev Clin Oncol (2017) 14(12):717–34. doi: 10.1038/nrclinonc.2017.101 28741618

[51] Gajewski TF Schreiber H Fu YX. Innate and Adaptive Immune Cells in the Tumor Microenvironment. Nat Immunol (2013) 14(10):1014–22. doi: 10.1038/ni.2703 PMC4118725 24048123

[52] Talmadge JE Gabrilovich DI. History of Myeloid-Derived Suppressor Cells. Nat Rev Cancer (2013) 13(10):739–52. doi: 10.1038/nrc3581 PMC4358792 24060865

[53] Dijkstra KK Cattaneo CM Weeber F Chalabi M van de Haar J Fanchi LF. Generation of Tumor-Reactive T Cells by Co-Culture of Peripheral Blood Lymphocytes and Tumor Organoids. Cell (2018) 174(6). doi: 10.1016/j.cell.2018.07.009 PMC6558289 30100188

[54] Grinberg-Bleyer Y Oh H Desrichard A Bhatt DM Caron R Chan TA. NF-κB C-Rel Is Crucial for the Regulatory T Cell Immune Checkpoint in Cancer. Cell (2017) 170(6). doi: 10.1016/j.cell.2017.08.004 PMC5633372 28886380

[55] Croasdell A Duffney PF Kim N Lacy SH Sime PJ Phipps RP. Pparγ and the Innate Immune System Mediate the Resolution of Inflammation. PPAR Res (2015) 2015:549691. doi: 10.1155/2015/549691 26713087 PMC4680113

[56] Du X Wen J Wang Y Karmaus PWF Khatamian A Tan H. Hippo/Mst Signalling Couples Metabolic State and Immune Function of CD8α Dendritic Cells. Nature (2018) 558(7708):141–5. doi: 10.1038/s41586-018-0177-0 PMC6292204 29849151

[57] Flecken T Schmidt N Hild S Gostick E Drognitz O Zeiser R. Immunodominance and Functional Alterations of Tumor-Associated Antigen-Specific CD8+ T-Cell Responses in Hepatocellular Carcinoma. Hepatology (Baltimore Md) (2014) 59(4):1415–26. doi: 10.1002/hep.26731 PMC4139003 24002931

[58] Geng J Yu S Zhao H Sun X Li X Wang P. The Transcriptional Coactivator TAZ Regulates Reciprocal Differentiation of T17 Cells and T Cells. Nat Immunol (2017) 18(7):800–12. doi: 10.1038/ni.3748 28504697

[59] Suh J-H Won KY Kim GY Bae GE Lim S-J Sung J-Y. Expression of Tumoral FOXP3 in Gastric Adenocarcinoma Is Associated With Favorable Clinicopathological Variables and Related With Hippo Pathway. Int J Clin Exp Pathol (2015) 8(11):14608–18. PMC4713570 26823784

[60] Ni X Tao J Barbi J Chen Q Park BV Li Z. YAP Is Essential for Treg-Mediated Suppression of Antitumor Immunity. Cancer Discov (2018) 8(8):1026–43. doi: 10.1158/2159-8290.CD-17-1124 PMC6481611 29907586

[61] Locati M Mantovani A Sica A. Macrophage Activation and Polarization as an Adaptive Component of Innate Immunity. Adv Immunol (2013) 120:163–84. doi: 10.1016/B978-0-12-417028-5.00006-5 24070384

[62] Chen Y Zhang S Wang Q Zhang X. Tumor-Recruited M2 Macrophages Promote Gastric and Breast Cancer Metastasis *via* M2 Macrophage-Secreted CHI3L1 Protein. J Hematol Oncol (2017) 10(1):36. doi: 10.1186/s13045-017-0408-0 28143526 PMC5286803

[63] Liang S Ma H-Y Zhong Z Dhar D Liu X Xu J. NADPH Oxidase 1 in Liver Macrophages Promotes Inflammation and Tumor Development in Mice. Gastroenterology (2019) 156(4):1156–72. doi: 10.1053/j.gastro.2018.11.019 PMC6409207 30445007

[64] Wei C Yang C Wang S Shi D Zhang C Lin X. M2 Macrophages Confer Resistance to 5-Fluorouracil in Colorectal Cancer Through the Activation of CCL22/PI3K/AKT Signaling. Onco Targets Ther (2019) 12:3051–63. doi: 10.2147/OTT.S198126 PMC6489624 31114248

[65] Prima V Kaliberova LN Kaliberov S Curiel DT Kusmartsev S. COX2/mPGES1/PGE2 Pathway Regulates PD-L1 Expression in Tumor-Associated Macrophages and Myeloid-Derived Suppressor Cells. Proc Natl Acad Sci USA (2017) 114(5):1117–22. doi: 10.1073/pnas.1612920114 PMC5293015 28096371

[66] Kimbrough-Allah MN Millena AC Khan SA. Differential Role of PTEN in Transforming Growth Factor β (TGF-β) Effects on Proliferation and Migration in Prostate Cancer Cells. Prostate (2018) 78(5):377–89. doi: 10.1002/pros.23482 PMC5820153 29341212

[67] Guo X Zhao Y Yan H Yang Y Shen S Dai X. Single Tumor-Initiating Cells Evade Immune Clearance by Recruiting Type II Macrophages. Genes Dev (2017) 31(3):247–59. doi: 10.1101/gad.294348.116 PMC5358722 28223311

[68] Gutmann DH Kettenmann H. Microglia/Brain Macrophages as Central Drivers of Brain Tumor Pathobiology. Neuron (2019) 104(3):442–9. doi: 10.1016/j.neuron.2019.08.028 PMC7288606 31697921

[69] Weng Y-S Tseng H-Y Chen Y-A Shen P-C Al Haq AT Chen L-M. MCT-1/miR-34a/IL-6/IL-6R Signaling Axis Promotes EMT Progression, Cancer Stemness and M2 Macrophage Polarization in Triple-Negative Breast Cancer. Mol Cancer (2019) 18(1):42. doi: 10.1186/s12943-019-0988-0 30885232 PMC6421700

[70] Huang Y-J Yang C-K Wei P-L Huynh T-T Whang-Peng J Meng T-C. Ovatodiolide Suppresses Colon Tumorigenesis and Prevents Polarization of M2 Tumor-Associated Macrophages Through YAP Oncogenic Pathways. J Hematol Oncol (2017) 10(1):60. doi: 10.1186/s13045-017-0421-3 28241877 PMC5329923

[71] Murakami S Shahbazian D Surana R Zhang W Chen H Graham GT. Yes-Associated Protein Mediates Immune Reprogramming in Pancreatic Ductal Adenocarcinoma. Oncogene (2017) 36(9):1232–44. doi: 10.1038/onc.2016.288 PMC5322249 27546622

[72] Wang G Lu X Dey P Deng P Wu CC Jiang S. Targeting YAP-Dependent MDSC Infiltration Impairs Tumor Progression. Cancer Discov (2016) 6(1):80–95. doi: 10.1158/2159-8290.CD-15-0224 26701088 PMC4707102

[73] White SM Murakami S Yi C. The Complex Entanglement of Hippo-Yap/Taz Signaling in Tumor Immunity. Oncogene (2019) 38(16):2899–909. doi: 10.1038/s41388-018-0649-6 PMC7567008 30617303

[74] Zhou X Li W Wang S Zhang P Wang Q Xiao J. YAP Aggravates Inflammatory Bowel Disease by Regulating M1/M2 Macrophage Polarization and Gut Microbial Homeostasis. Cell Rep (2019) 27(4):1176–89. doi: 10.1016/j.celrep.2019.03.028 31018132

[75] Yang W Yang S Zhang F Cheng F Wang X Rao J. Influence of the Hippo-YAP Signalling Pathway on Tumor Associated Macrophages (TAMs) and Its Implications on Cancer Immunosuppressive Microenvironment. Ann Trans Med (2020) 8(6):399. doi: 10.21037/atm.2020.02.11 PMC7186717 32355843

[76] Janse van Rensburg HJ Azad T Ling M Hao Y Snetsinger B Khanal P. The Hippo Pathway Component TAZ Promotes Immune Evasion in Human Cancer Through PD-L1. Cancer Res (2018) 78(6):1457–70. doi: 10.1158/0008-5472.CAN-17-3139 29339539

[77] Lee BS Park DI Lee DH Lee JE Yeo MK Park YH. Hippo Effector YAP Directly Regulates the Expression of PD-L1 Transcripts in EGFR-TKI-Resistant Lung Adenocarcinoma. Biochem Biophys Res Commun (2017) 491(2):493–9. doi: 10.1016/j.bbrc.2017.07.007 28684311

[78] Tung JN Lin PL Wang YC Wu DW Chen CY Lee H. PD-L1 Confers Resistance to EGFR Mutation-Independent Tyrosine Kinase Inhibitors in Non-Small Cell Lung Cancer *via* Upregulation of YAP1 Expression. Oncotarget (2018) 9(4):4637–46. doi: 10.18632/oncotarget.23161 PMC5797002 29435131

[79] Restifo NP Smyth MJ Snyder A. Acquired Resistance to Immunotherapy and Future Challenges. Nat Rev Cancer (2016) 16(2):121–6. doi: 10.1038/nrc.2016.2 PMC6330026 26822578

[80] Sharma P Hu-Lieskovan S Wargo JA Ribas A. Primary, Adaptive, and Acquired Resistance to Cancer Immunotherapy. Cell (2017) 168(4):707–23. doi: 10.1016/j.cell.2017.01.017 PMC5391692 28187290

[81] Wagenblast E Soto M Gutierrez-Angel S Hartl CA Gable AL Maceli AR. A Model of Breast Cancer Heterogeneity Reveals Vascular Mimicry as a Driver of Metastasis. Nature (2015) 520(7547):358–62. doi: 10.1038/nature14403 PMC4634366 25855289

[82] Kim J Kim YH Kim J Park DY Bae H Lee DH. YAP/TAZ Regulates Sprouting Angiogenesis and Vascular Barrier Maturation. J Clin Invest (2017) 127(9):3441–61. doi: 10.1172/JCI93825 PMC5669570 28805663

[83] Wang X Freire Valls A Schermann G Shen Y Moya IM Castro L. YAP/TAZ Orchestrate VEGF Signaling During Developmental Angiogenesis. Dev Cell (2017) 42(5):462–78.e7. doi: 10.1016/j.devcel.2017.08.002 28867486

[84] Ferrara N. VEGF as a Therapeutic Target in Cancer. Oncology (2005) 69(Suppl 3):11–6. doi: 10.1159/000088479 16301831

[85] Chappell JC Wiley DM Bautch VL. Regulation of Blood Vessel Sprouting. Semin Cell Dev Biol (2011) 22(9):1005–11. doi: 10.1016/j.semcdb.2011.10.006 PMC4521217 22020130

[86] Cebe-Suarez S Zehnder-Fjallman A Ballmer-Hofer K. The Role of VEGF Receptors in Angiogenesis; Complex Partnerships. Cell Mol Life Sci (2006) 63(5):601–15. doi: 10.1007/s00018-005-5426-3 PMC2773843 16465447

[87] Azad T Janse van Rensburg HJ Lightbody ED Neveu B Champagne A Ghaffari A. A LATS Biosensor Screen Identifies VEGFR as a Regulator of the Hippo Pathway in Angiogenesis. Nat Commun (2018) 9(1):1061. doi: 10.1038/s41467-018-03278-w 29535383 PMC5849716

[88] Elaimy AL Mercurio AM. Convergence of VEGF and YAP/TAZ Signaling: Implications for Angiogenesis and Cancer Biology. Sci Signal (2018) 11(552). doi: 10.1126/scisignal.aau1165 PMC6525620 30327408

[89] Kim W Khan SK Gvozdenovic-Jeremic J Kim Y Dahlman J Kim H. Hippo Signaling Interactions With Wnt/beta-Catenin and Notch Signaling Repress Liver Tumorigenesis. J Clin Invest (2017) 127(1):137–52. doi: 10.1172/JCI88486 PMC5199712 27869648

[90] Young K Tweedie E Conley B Ames J FitzSimons M Brooks P. BMP9 Crosstalk With the Hippo Pathway Regulates Endothelial Cell Matricellular and Chemokine Responses. PloS One (2015) 10(4):e0122892. doi: 10.1371/journal.pone.0122892 25909848 PMC4409298

[91] Mo JS Meng Z Kim YC Park HW Hansen CG Kim S. Cellular Energy Stress Induces AMPK-Mediated Regulation of YAP and the Hippo Pathway. Nat Cell Biol (2015) 17(4):500–10. doi: 10.1038/ncb3111 PMC4380774 25751140

[92] Wang W Xiao ZD Li X Aziz KE Gan B Johnson RL. AMPK Modulates Hippo Pathway Activity to Regulate Energy Homeostasis. Nat Cell Biol (2015) 17(4):490–9. doi: 10.1038/ncb3113 PMC4380807 25751139

[93] Totaro A Castellan M Battilana G Zanconato F Azzolin L Giulitti S. YAP/TAZ Link Cell Mechanics to Notch Signalling to Control Epidermal Stem Cell Fate. Nat Commun (2017) 8:15206. doi: 10.1038/ncomms15206 28513598 PMC5442321

[94] Lu J Zhou Y Hu T Zhang H Shen M Cheng P. Notch Signaling Coordinates Progenitor Cell-Mediated Biliary Regeneration Following Partial Hepatectomy. Sci Rep (2016) 6:22754. doi: 10.1038/srep22754 26951801 PMC4782135

[95] Conti B Slemmons KK Rota R Linardic CM. Recent Insights Into Notch Signaling in Embryonal Rhabdomyosarcoma. Curr Drug Targets (2016) 17(11):1235–44. doi: 10.2174/1389450116666150907105756 26343114

[96] Ignatius MS Hayes MN Lobbardi R Chen EY McCarthy KM Sreenivas P. The NOTCH1/SNAIL1/MEF2C Pathway Regulates Growth and Self-Renewal in Embryonal Rhabdomyosarcoma. Cell Rep (2017) 19(11):2304–18. doi: 10.1016/j.celrep.2017.05.061 PMC5563075 28614716

[97] Slemmons KK Crose LES Riedel S Sushnitha M Belyea B Linardic CM. A Novel Notch-YAP Circuit Drives Stemness and Tumorigenesis in Embryonal Rhabdomyosarcoma. Mol Cancer Res (2017) 15(12):1777–91. doi: 10.1158/1541-7786.MCR-17-0004 PMC5755394 28923841

[98] Park HW Kim YC Yu B Moroishi T Mo JS Plouffe SW. Alternative Wnt Signaling Activates YAP/TAZ. Cell (2015) 162(4):780–94. doi: 10.1016/j.cell.2015.07.013 PMC4538707 26276632

[99] Rosenbluh J Nijhawan D Cox AG Li X Neal JT Schafer EJ. Beta-Catenin-Driven Cancers Require a YAP1 Transcriptional Complex for Survival and Tumorigenesis. Cell (2012) 151(7):1457–73. doi: 10.1016/j.cell.2012.11.026 PMC3530160 23245941

[100] Tao J Calvisi DF Ranganathan S Cigliano A Zhou L Singh S. Activation of Beta-Catenin and Yap1 in Human Hepatoblastoma and Induction of Hepatocarcinogenesis in Mice. Gastroenterology (2014) 147(3):690–701. doi: 10.1053/j.gastro.2014.05.004 24837480 PMC4143445

[101] Ma LG Bian SB Cui JX Xi HQ Zhang KC Qin HZ. LKB1 Inhibits the Proliferation of Gastric Cancer Cells by Suppressing the Nuclear Translocation of Yap and Beta-Catenin. Int J Mol Med (2016) 37(4):1039–48. doi: 10.3892/ijmm.2016.2494 26936013

[102] Krishnamurthy N Kurzrock R. Targeting the Wnt/beta-Catenin Pathway in Cancer: Update on Effectors and Inhibitors. Cancer Treat Rev (2018) 62:50–60. doi: 10.1016/j.ctrv.2017.11.002 29169144 PMC5745276

[103] Choi W Kim J Park J Lee DH Hwang D Kim JH. YAP/TAZ Initiates Gastric Tumorigenesis *via* Upregulation of MYC. Cancer Res (2018) 78(12):3306–20. doi: 10.1158/0008-5472.CAN-17-3487 29669762

[104] Deng L Meng T Chen L Wei W Wang P. The Role of Ubiquitination in Tumorigenesis and Targeted Drug Discovery. Signal Transduct Target Ther (2020) 5(1):11. doi: 10.1038/s41392-020-0107-0 32296023 PMC7048745

[105] Dey A Varelas X Guan KL. Targeting the Hippo Pathway in Cancer, Fibrosis, Wound Healing and Regenerative Medicine. Nat Rev Drug Discov (2020) 19(7):480–94. doi: 10.1038/s41573-020-0070-z PMC7880238 32555376

